# Is ‘legal empowerment of the poor’ relevant to people with disabilities in developing countries? An empirical and normative review

**DOI:** 10.3402/gha.v6i0.22854

**Published:** 2013-11-15

**Authors:** Johan Borg, Anna-Karin Bergman, Per-Olof Östergren

**Affiliations:** 1 Division of Social Medicine and Global Health, Lund University, Malmö, Sweden; 2 Centre for Sustainability Studies, Lund University, Sweden

**Keywords:** developing country, disability, legal empowerment, low-income country, poverty

## Abstract

**Background:**

Legal empowerment of the poor is highly relevant to public health as it aims to relieve income poverty, a main determinant of health. The Commission on Legal Empowerment of the Poor (CLEP) has proposed legal empowerment measures in the following four domains: access to justice and the rule of law, property, labor, and business rights. Despite being overrepresented among the poor, CLEP has not explicitly considered the situation of people with disabilities.

**Objectives:**

To examine the empirical evidence for the relevance of the CLEP legal empowerment measures to people with disabilities in low- and lower middle-income countries, and to evaluate the extent to which the Convention on the Rights of Persons with Disabilities (CRPD) addresses those measures.

**Methods:**

Critical literature review of empirical studies and a checklist assessment of the CRPD.

**Results:**

Fourteen included articles confirm that people with disabilities experience problems in the domains of access to justice and the rule of law, labor rights, and business rights. No texts on property rights were found. Evidence for the effectiveness of the proposed measures is insufficient. Overall, the CRPD fully or partially supports two-thirds of the proposed measures (seven out of nine measures for access to justice and the rule of law, none of the five measures for property rights, all seven measures for labor rights, and six out of nine measures for business rights).

**Conclusions:**

Although most of the domains of the CLEP legal empowerment measures are relevant to people with disabilities from both empirical and normative perspectives, it is uncertain whether the devised measures are of immediate relevance to them. Further research is warranted in this regard.

Legal empowerment may be understood as the use of law specifically to strengthen the disadvantaged (1). Legal empowerment of the poor is highly relevant to public health as it aims at relieving income poverty, a main determinant of health. Considering the links between income poverty, health, and disability in low-income countries (2, 3), legal empowerment of the poor may be of particular relevance to people with disabilities in these countries.

Legal empowerment of the poor can be seen as a broader tool as it also addresses other important social determinants of health, such as improving the circumstances in which people live and work (4). To improve health and health equity, the inequitable distribution of and access to power, wealth, and social resources need to be tackled. Unfair distribution of and access to these means are often tolerated or promoted by social norms, policies, and practices. Potentially, every aspect of government and the economy affect health and health equity. Immediate risks for ill health, which are distributed in an inequitable way, are determined by factors at a higher societal level, for example, the political, judicial, and economic system, which reflects the specific power relations of that society. Besides bringing health benefits in the form of new technologies, goods and services, and improved standards of living, the market place can also generate negative conditions for health, for example, unhealthy working conditions, environmental pollution, and economic inequalities (5, 6).

Hosted by the United Nations Development Programme (UNDP), the International Commission on Legal Empowerment of the Poor (CLEP) was formed in 2005 to focus on the link among exclusion, poverty, and the law. In its final report entitled ‘Making the Law Work for Everyone’, CLEP estimated that at least four billion people are excluded from the rule of law (7). By giving poor women and men access to justice, and by underpinning and enabling certain rights, the Report argues that they can be empowered to change their lives for the better. Despite poverty being the reality for many of the world's one billion people with disabilities (3), the Report does not explicitly relate to them. Therefore, drawing from available evidence, this article intends to explore the relevance of the recommendations made by the Report for reducing poverty – and thereby promote health – among people with disabilities living in countries where incomes in general are low.

## Legal empowerment

In the Report, legal empowerment is viewed as a process through which poor people become protected and are enabled to use the law to advance their rights and their interests. It involves them realizing their rights and reaping resulting opportunities through public support as well as efforts of themselves, their supporters, and networks (7).

Since legal empowerment (as a political concept) was first coined by the Asian Development Bank in the Asia Foundation Report from 2001 (8), it has been used and defined by a number of different institutions such as the Carnegie Endowment for International Peace (9). The concept gained particular momentum through the Report, which perceives legal empowerment as a ‘global social contract’ primarily encompassing access to justice and the rule of law, property rights, labor rights, and business rights (10). Property rights, labor rights, and so-called business rights have been singled out as particular vital empowerment domains, mostly due to their connection to economic agency; an area essential for development in general and poverty reduction in particular. Together with access to justice and the rule of law, these three domains are labeled ‘pillars of legal empowerment’ (the Pillars) (7). (CLEP recognizes that ‘business rights’ is not a human rights category *per se* and does not advocate for it to be established as such, but acknowledges that this category is ‘derived from [other] existing rights related to doing business of the individual’ (7, p. 5).)

Legal empowerment is claimed to highlight a ‘forgotten’ dimension of poverty not covered by the Millennium Development Goals (MDGs): that poverty is an outcome of legal deprivation, that is, there is a lack of legal recognition and protection of rights for excluded groups (11). In this context, the primary function of legal empowerment of the poor is to advance ‘the process of systemic change through which the poor and excluded become able to use the law, the legal system and legal services, to protect and advance their rights and interests as citizens and economic actors’ (7, p. 3). Legal empowerment does not consist of a single strategy, but it usually targets disadvantaged populations, and focuses on the use of law, the legal system and legal services in broad terms as instruments for development, sustainable livelihood, and poverty reduction for the disadvantaged (12).

The Pillars are based on a human rights-based approach and draws from the Universal Declaration of Human Rights (UDHR) (13). The first pillar *access to justice and the rule of law* follows from UDHR Articles 6 and 7, the second pillar *property rights* finds its basis in UDHR Article 17, and the third pillar *labor rights* is based on UDHR Article 23. The fourth pillar *business rights* is a composite of rights of people to engage in economic activity and market transactions. They include the right of people to start a legally recognized business without arbitrarily applied regulations or discrimination in the application of norms and procedures. It focuses on removing unnecessary barriers that limit economic opportunities and on protecting the investments that people make in their enterprises. Business activities are an expression of an entire class of liberties, namely freedom of association, freedom of movement, freedom to develop one's own talents, and freedom to exchange legitimately acquired goods and services (7).

The Report acknowledges that poverty eradication cannot be accomplished through legal empowerment alone. However, although not sufficient in itself, legal empowerment is regarded as a necessary remedy for inclusion economically, politically, socially, and morally (7). Considering its recommendations being top-down, impractical and unrealistic, the Report has received criticism (14, 15). Moreover, it has been argued that legal empowerment of the poor needs to be linked with general development experience, and that the Report fails to justify its policy agenda with empirical data (16, 17). Despite its shortcomings, it has been claimed that the Report has contributed to the international development community by focusing attention on legal needs of poor populations (15).

## Disability

People with disabilities in low-income countries are often deprived of basic human rights and overrepresented among the poor (3, 18–20). Compared to those without disabilities, they generally have lower employment rates and educational attainment. There is a growing evidence base that indicates substantial links among poverty, health, and disability in these countries (2, 3).

The International Classification of Functioning, Disability and Health (ICF) intends to encompass all aspects of human health and some health-relevant components of well-being. It describes them in terms of health domains and health-related domains. Examples of health domains include seeing, hearing, walking, learning and remembering, while examples of health-related domains include education, work and employment, and transportation (21).

According to the ICF, disability is understood as the negative aspects of the dynamic interaction between an individual's health conditions (diseases, disorders, injuries, traumas, etc.), personal factors (gender, race, age, fitness, lifestyle, habits, social background, education, profession, etc.) and environmental factors (elements of the physical, social, and attitudinal environments). Disability is used as an umbrella term for impairments, activity limitations, and participation restrictions (21).

The environmental factors in the ICF focus on two different levels: individual and societal. At the individual level, the factors include physical and material features of, and direct contacts with others in the immediate environment of an individual (e.g. home, school, and workplace). At the societal level, the factors encompass formal and informal social structures, services, and overarching approaches or systems in the community or society (e.g. organizations and services related to the work environment, government agencies, communication and transportation services, informal social networks, laws, regulations, and attitudes). Society may hinder an individual's performance by creating barriers (e.g. inaccessible buildings) or by not providing facilitators (e.g. unavailability of wheelchairs, hearing aids or other assistive technology) (21).

Both elimination of barriers and provision of facilitators may be addressed through adoption and implementation of appropriate legislation, which is thus considered an environmental factor at societal level (21). A key example is the Convention on the Rights of Persons with Disabilities (CRPD), which was adopted in late 2006 to promote, protect, and ensure the enjoyment of all human rights and fundamental freedoms by all people with disabilities (22). Regarded as having empowered the world's largest minority, it focuses on the actions States must take to ensure that people with disabilities can enjoy civil, cultural, economic, political, and social rights on an equal basis with others (22, 23).

## Rights, health, and development

Legal empowerment is a strategy to facilitate human development through realization of a selected number of human rights. Therefore, it has been suggested that this approach is viewed as a subset of the broader human rights-based approach to development and poverty reduction (16).

Human development has increasingly been seen as expanding economic, social, cultural, and political freedoms rather than economic growth alone (24). Through his capability approach, Sen connects human development to human rights (25, 26). He suggests that human rights can be seen as entitlements to capabilities (things a person is substantively free to do or be) and that expanding capabilities is development (26, 27). This expansion can be accomplished by providing access to such basic capabilities as healthcare, education, and the labor market, which are all integral parts of the Pillars (7, 28). Equity-driven development should be viewed as a contribution to sustainability, as redistribution to the poor in the form of improving their health, education, and nutrition will have impacts on the current as well as future generations (29).

It is well known that health is both a consequence of and a factor contributing to the development of societies. Health is thereby central to sustainable development and its components of social, economic, and ecological sustainability. Unequally distributed resources in a society have a major impact on health and its social distribution. Equity, therefore, appears to be a key for sustainability defined as the survival and well-being of humans (30). Thus, considering legal empowerment as an equalizer of poverty alleviating opportunities, it plays a role for more sustainable and healthier societies.

## Aim and objectives

The purpose of this study has been to contribute to expanding the knowledge about strategies to reduce poverty among people with disabilities in low- and lower middle-income countries from a rights perspective, while keeping in mind potential positive health and sustainability outcomes. Considering that the CLEP recommendations neither does explicitly address the situation of people with disabilities in these countries nor does it relate to the legal empowerment enshrined in the CRPD, the specific aim was to explore the relevance of the Pillars to them. To fulfill this aim, the objectives have been:

1. to examine the evidence base for the relevance of the Pillars to people with disabilities in low- and lower middle-income countries and
2. to evaluate the extent to which the CRPD addresses the Pillars.

Consequently, the study set out to answer the following research questions:

1. What is the empirical evidence for problems related to the Pillars among people with disabilities in low- and lower middle-income countries and for solutions to those problems?
2. To what extent does the CRPD provide normative support for the Pillars?

## Methods

The first research question was addressed through a critical review and the second one through a checklist assessment.

### Critical review

To examine the evidence base, qualitative and quantitative research articles were searched in the following databases on October 11, 2012: CINAHL, HeinOnline, PubMed, SocIndex, Web of Science. Using Boolean technique, the following combination of search terms was used in each database: (disab* OR handic* OR impair*) AND (developing countr* OR low income countr* OR low income econom* OR lower middle income countr* OR lower middle income econom*) AND (legal* empower* OR poverty OR poor OR human right* OR labour OR labor OR employment OR work OR propert* OR rule of law OR justice OR exclusion OR (freedom AND (produce OR trade OR consume OR exchange))). Although health, education, and social security issues are included in the domains of the Pillars, such search terms were not included in this search.

An article was included if it presents original qualitative or quantitative research findings about the situation of people with disabilities with regard to the Pillars in a low- or lower middle-income countries, as defined by the World Bank as of July 2012 (31), and was published in a peer-reviewed journal in 2002 or later. Studies covering low- or lower middle-income countries were included. Studies in upper middle countries were included if they also involved low- or lower middle-income countries. Studies in upper middle-income countries only were excluded. Articles not dealing with long-term physical, sensory, mental, or intellectual disabilities were excluded. Without language restriction searches were performed in titles and abstracts, in databases where such limits were possible, or in article texts.

**Fig. 1:**
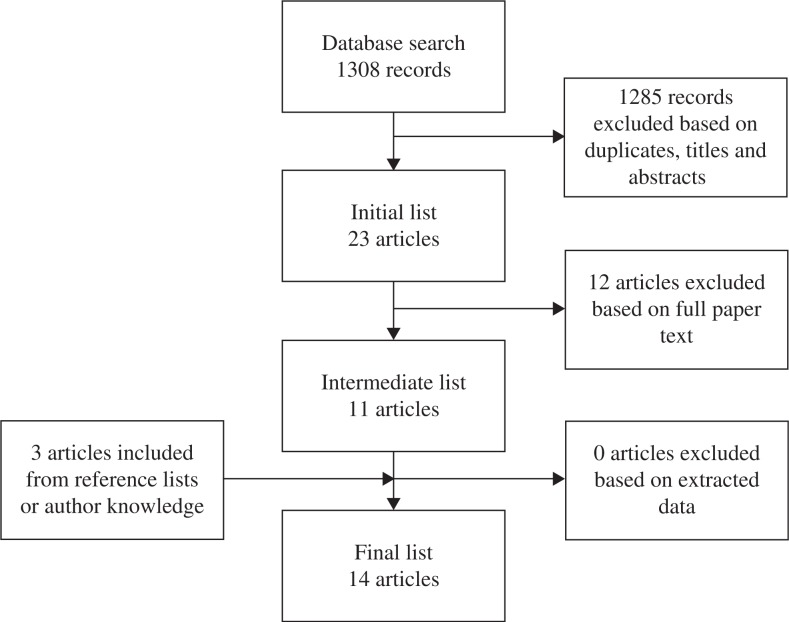
Process of selecting articles.

An overview of the process of selecting articles is given in Fig. 1. The titles and abstracts of a total of 1,308 hits generated by searches in the databases were screened manually. A search record by database is presented in Table 1. Duplicates and articles not meeting the criteria were excluded, resulting in an initial list of 23 articles potentially relevant to this study. The full articles in the initial list were studied and articles not meeting all criteria were excluded resulting in an intermediate list of 11 articles. The reference lists were searched for any additional research meeting the selection criteria. This added another two articles. From the authors’ knowledge, one accepted but not yet published article was added. The selected articles in the intermediate list and the added articles were reviewed and a data extraction form was completed for each of them. Based on extracted data, the authors discussed the articles and determined that all 14 articles would be included in the final list.

**Table 1:** Results of database searches

Database	Records
CINAHL	37
HeinOnline	7
PubMed	339
SocIndex	32
Web of Science	893

Data from articles in the final list were analyzed and the findings categorized according to four domains corresponding to the Pillars. As the studies varied widely in designs and methods, a complete evaluation of the strength of the evidence was not carried out. However, when applicable, information about sample size, study design, and instrumentation were compiled.

### Checklist assessment

In order to evaluate the extent to which the CRPD addresses the Pillars, a checklist assessment was undertaken. This method may be used when evaluating, for example, health policies to know whether a policy adequately addresses various content issues and have included appropriate actions (32, 33). First, a checklist based on the measures and liberties proposed in the Report was developed. The checklist comprises 30 items in four domains, which correspond to the four Pillars. Second, the content of the CRPD was assessed using the checklist. The extent to which the CRPD addresses each legal empowerment measure was rated *Addressed not at all*, *Addressed partly* or *Addressed fully*. Only specific CRPD measures were considered. Thus, general stipulations such as ‘take all necessary measures’ or ‘take appropriate steps’ were not taken into account in this evaluation.

## Results

The findings from the critical review and the checklist assessment are presented in the following sections.

### Overview of review findings

As described above, a total of 14 articles were included in the final list. In Table 2, the representation of economies and regions, according to World Bank definitions, of the reviewed articles is given. Ten of them included low-income countries and nine included lower middle-income countries. Articles with countries located in South Asia and sub-Saharan Africa were most frequent (10 and 8 articles, respectively), while there were fewer articles concerning countries from other regions.

**Table 2:** Representation of economies and regions

World Bank economies and regions	No. of articles
Low income	10
Lower middle-income	9
Upper middle-income	3
East Asia and Pacific	3
Europe and Central Asia	1
Latin America and Caribbean	2
Middle East and North Africa	1
South Asia	10
Sub-Saharan Africa	8

In Table 3, an overview is given of the objectives, designs, instruments, settings, samples, and main findings of the studies reported in the reviewed articles. They report the results of seven quantitative and four qualitative cross-sectional studies, and three case studies. Five studies used instruments based on WHO resources, two studies applied instruments based on World Bank resources and one study used a national survey instrument.

**Table 3:** Overview of included articles

First author, year, and reference	Objective	Study design, instrument, and setting	Sample	Main findings
Alade 2004 (43)	To describe a community-based vocational rehabilitation (CBVR) program and its outcomes.	Case study. Nigeria.	A CBVR program with a total of 155 participants with various disabilities over 8 years.	More than 90% of the participants were gainfully employed. About 10% of the trainees had benefited from a revolving loan scheme.
Borg 2012 (45)	To explore the relation between assistive technology use and enjoyment of human rights.	Quantitative cross-sectional. Questionnaire based on ICF. Bangladesh.	Hearing impairment: 136 people using hearing aids, 149 not using hearing aids. Ambulatory impairment: 149 people using wheelchairs, 149 people not using wheelchair.	Hearing aid use was positively associated with access to medical care, physical, and mental health, literacy, education, participation in school, participation in work, participation in using public transportation, and ambulatory performance. Working status did not differ statistically significantly between users and non-users of hearing aids. Wheelchair use was statistically significantly associated with better ambulatory performance and lower likelihood of working. Physical accessibility to working places and duration of wheelchair use was statistically significantly associated with higher participation in work and positive work status, respectively.
Dhungana 2006 (36)	To explore and discuss the lives of women with disabilities and analyze how external factors put these women at the lowest level of existence.	Qualitative cross-sectional (interview). Nepal.	30 women with physical disabilities.	Women had no or little education, were discriminated against when it comes to jobs and were excluded from employment. Women got less advantage of vocational training programs for people with disabilities. Men got training on skills in high demand. The built environment is not accessible. Inaccessible buildings, including toilets, make it impossible to keep a job or to look for training and jobs. Public transportation services were inaccessible. (Women are denied equal inheritance of property.)
Drew 2011 (34)	To review the evidence for the types of human rights violations experienced by people with mental and social disabilities in low-income and middle-income countries as well as strategies to prevent these violations and promote human rights.	Qualitative cross-sectional (questionnaire, literature review). Questionnaire partly based on WHO Quality Rights Assessment Tool. Belize, Bosnia and Herzegovina, Egypt, Georgia, Ghana, Indonesia, Jordan, Kenya, Lithuania, Mexico, Nepal, Paraguay, Peru, South Africa, Sri Lanka, Tajikistan, West Bank and Gaza, Zambia.	51 people with mental and psychosocial disabilities. Literature in English (UN publications, non-governmental organization reports, press reports, academic literature)	Stigma and discrimination lead to violations of human rights, including civil, cultural, economic, political, and social rights. Denial of people's rights to exercise legal capacity and discrimination in employment are central issues.
Fritz 2009 (35)	To examine the inclusive dimension of poverty-reduction strategy (PRS) processes, to review the relationship between poverty and disability in three projects, to describe the three projects and to analyze their successes and challenges.	Case studies. Cambodia, Tanzania, Vietnam.	3 PRS projects	The PRS process provides a framework for capacity development of disabled peoples’ organizations and for raising awareness on disabilities among government and civil society. The public and authorities perceived people with disabilities differently and acknowledged their rights and needs. In Cambodia, community bodies signalled that they plan to include disability aspects in their development plans and people with disabilities were called to participate in vocational training programs. It is important to create sustainable structures of empowerment.
Hosain 2002 (39)	To examine the impact of disability on the quality of life of people with disabilities.	Quantitative cross-sectional. ICF, SF-36. Bangladesh.	162 people with disabilities	More than 70% of 57 respondents aged 5–30 years had been unable to attend or compelled to leave school due to disability. About four out of five men aged above 12 responded that disability had negative consequence on their employment, including lower salary than non-disabled, forced to change employment or being unemployed.
Kiani 2009 (37)	To analyze the gap in current and desired life situations of women with disabilities.	Qualitative cross-sectional (focus groups, interviews and participant observation). Cameroon.	Focus groups: 24 (15 + 9) Interviews: 12	Difficulty in obtaining employment due to discrimination by employers or lack of education. Difficult to move around and reach markets, workplaces, etc. No public or accessible transportation system. Negative attitudes when attempting to use taxi or using the road. Desire to learn income-generating skills and entrepreneurships. Need for capital to run and maintain businesses.
Mitra 2008 (38)	To explain employment differences between males with and without disabilities.	Quantitative cross-sectional. World Bank Village Disability Survey. India.	262 men with disabilities, 980 men without disabilities.	The employment rate for men without disabilities was higher than for men with disabilities. The gap was not explained by differences in human capital and productivity. It may result from differential returns to characteristics and from discrimination.
Mitra 2013 (40)	To draw an economic profile of persons with disabilities.	Quantitative cross-sectional. World Health Survey. Bangladesh, Brazil, Burkina Faso, Dominican Republic, Ghana, Kenya, Lao PDR, Malawi, Mauritius, Mexico, Pakistan, Paraguay, Philippines, Zambia, Zimbabwe.	All countries together: 7,443 persons with disabilities, 84,381 persons without disabilities.	In 14 countries, people with disabilities had completed primary school to a statistically significantly less degree than people without disabilities. In 9 countries, people with disabilities were employed to a statistically significantly less degree than people without disabilities.
Rischewski 2008 (42)	To assess the association between poverty and musculoskeletal impairment (MSI).	Quantitative cross-sectional. Questionnaire based on World Bank's Living Standards Measurement Study. Rwanda.	345 persons with MSI, 532 controls	Adults with MSI were more likely to have no employment. Children with MSI were less likely to attend school.
Savill 2003 (46)	To identify barriers experienced by people with disabilities when using public transport services in urban areas of the developing world.	Qualitative cross-sectional (interview, focus groups). India, Malawi, Mozambique, South Africa.	Up to 150 people with disabilities in each country.	Three types of barriers to accessibility were identified: structural, social, and psychological.
Trani 2012 (28)	To compare data collected from household surveys and to explore the potential link between multidimensional poverty and disability.	Quantitative cross-sectional. Questionnaire partly based on ICF. Afghanistan, Zambia.	Afghanistan: 641 persons with disabilities, 903 controls. Zambia: 1,845 persons with disabilities, 1,643 controls.	People with disabilities have lower access to education and labor market. To some extent they also have less access to healthcare services. Women with disabilities had a very low likelihood to have had access to school. Persons with cognitive, mental or multiple disabilities were the least likely to work.
Uppal 2007 (41)	To explore the relationship between the health status of the elderly and their labor market participation.	Quantitative cross-sectional. 1995/96 National Sample Survey. India.	32,338 aged 60 years and above, nationally representative	Disabilities and chronic illnesses have a negative association with the probability of working, the effect being stronger in rural areas.
Werner 2009 (44)	To present corporate social responsibility (CSR) projects that are designed to reduce social exclusion among marginalized populations and to explore whether CSR initiatives can increase economic and social capabilities to reduce exclusion.	Case studies (interviews, literature). Bangladesh.	Purposive selection of CSR examples, including one targeting, among others, women with disabilities.	One example of the CSR initiatives increases job skills and employment opportunities for women with disabilities. More than 80 out of 1,800 women have a disability.

Column 4 of Table 4 indicates which of the 30 legal empowerment measures are addressed by which reviewed article.

**Table 4:** What legal empowerment measures of the CLEP pillars are addressed by which CRPD articles and reviewed articles.

	CRPD articles addressing the measure^1^		
		
Legal empowerment measures	Fully	Partly	Reviewed articles addressing the measure
Domain: access to justice and the rule of law (7, pp. 5–6)			
1. Ensure that every person with disability has the right to legal identity and is registered at birth.	12(1–2); 18(2)		(34)^2^
2. Repeal or modify laws and regulations that are biased against the rights, interests, and livelihoods of people with disabilities.	4(1.a–c,e)		(34, 35)
3. Facilitate the creation of state and civil society organizations and coalitions, including paralegals who work in the interest of people with disabilities.		4(3);33(1,3)	(35)
4. Establish a legitimate state monopoly on the means of coercion, through, for example, effective and impartial policing.		14(1.b,2); 15(2);16(4)	
5. Recognize and integrate customary and informal legal procedures with which people with disabilities are already familiar (regarding the formal judicial system, land administration systems and relevant public institutions).			
6. Encourage courts to give due consideration to the interests of people with disabilities.	13		
7. Support mechanisms for alternative dispute resolution.			
8. Foster and institutionalize access to legal services so that people with disabilities will know about laws and be able to take advantage of them.	12(3); 13; 16(2)		
9. Support concrete measures for the legal empowerment of women, minorities, refugees, internally displaced persons and indigenous people with disabilities.		6(2)	
Domain: property rights (7, p. 7)			
10. Promote efficient governance of individual and collective property in order to integrate the extralegal economy into the formal economy and ensure it remains easily accessible to people with disabilities.			
11. Ensure that all property recognized in each nation is legally enforceable by law and that all owners with disabilities have access to the same rights and standards.			(36)^2^
12. Create a functioning market for the exchange of assets that is accessible, transparent, and accountable.			
13. Broaden the availability of property rights, including tenure security, through social and other public policies, such as access to housing, low interest loans and the distribution of state land.			(36)^2^
14. Promote an inclusive property rights system that will automatically recognize real and immoveable property bought by men as the co-property of their wives or common-law partners.			(36)^2^
Domain: labor rights (7, pp. 7–8)			
15. Respect, promote, and realize freedom of association so that the identity, voice, and representation of the working people with disabilities can be strengthened in the social and political dialog about reform and its design.	27(1.c); 29(b)		
16. Improve the quality of labor regulation and the functioning of labor market institutions, thereby creating synergy between the protection and productivity of people with disabilities.	27		
17. Ensure effective enforcement of a minimum package of labor rights for workers with disabilities and enterprises in the informal economy that upholds and goes beyond the Declaration of Fundamental Principles and Rights at Work.		27^3^	
18. Increase access to employment opportunities for people with disabilities in the growing and more inclusive market economy.		27	(28, 34–45)
19. Expand social protection for workers with disabilities in the event of economic shocks and structural changes.		27(1.e); 28(2)	
20. Promote measures that guarantee access to medical care, health insurance and pensions for people with disabilities.	25; 26; 28(2.e)		(28, 45)
21. Ensure that legal empowerment drives gender equality among people with disabilities.	6(2)		
Domain: business rights (7, pp. 8–9, 31)			
22. Guarantee basic business rights for people with disabilities; including the right to vend, to have a workspace, and to have access to necessary infrastructure and services (shelter, electricity, water, sanitation).		26(1.b); 27(f)	
23. Strengthen effective economic governance that makes it easy and affordable for people with disabilities to set up and operate a business, to access markets and to exit a business if necessary.		19(b); 26(1.b); 27(f)	
24. Expand the definition of ‘legal person’ to include legal liability companies that allow owners to separate their business and personal assets, thus enabling prudent risk taking.			
25. Promote inclusive financial services that offer entrepreneurs with disabilities savings, credit, insurance, pensions and other tools for risk management.		19(c); 27(1.f)	(37, 43)
26. Expand access to new business opportunities through specialized programs to familiarize entrepreneurs with disabilities with new markets and help them comply with regulations and requirements, and that support backward and forward linkages between larger and smaller firms.			(43)
27. Ensure freedom of association of people with disabilities.		27(1.c); 29(b)	
28. Ensure freedom of movement of people with disabilities.	18(1); 20		(36, 37, 45, 46)
29. Ensure freedom of people with disabilities to develop one's owns talents.	30(2)		(28, 35–37, 39, 40, 42, 44, 45)
30. Ensure freedom of people with disabilities to exchange legitimately acquired goods and services.			

1 Numbers in brackets refer to specific sections of the mentioned CRPD article.

2 The reviewed article addresses the measure to a limited extent.

3 CRPD does address labor rights for workers with disabilities but not explicitly for enterprises in the informal economy.

### Access to justice and the rule of law

Two of the reviewed articles present findings related to access to justice and the rule of law; one of them attempts to describe the situation, while the other reports on an intervention and its outcomes.

In a consultation with 51 people with mental and psychosocial disabilities from 18 low- and middle-income countries, Drew et al. (34) found that stigma and discrimination lead to violations of human rights, particularly denial of the right to exercise legal capacity.

Fritz et al. (35) have studied the inclusive dimension of poverty-reduction strategy processes in three countries. They conclude that bringing people with disabilities into this process can be a powerful tool for improving their situation, as it empowers people with disabilities, develops the capacities of their organizations, and raise awareness about their situation among government and civil society. In Cambodia, community bodies planned to include disability aspects in their local development plans (35).

### Property rights

None of the reviewed articles explicitly addresses property rights. In her qualitative study of the situation of 30 Nepalese women with physical disabilities, Dhungana (36) noted that they were denied the right of equal inheritance of property irrespective of disability status, although disability brings discrimination one step further.

### Labor rights

Labor rights issues were addressed by 13 of the reviewed articles. Nine of them describe the situation of people with disabilities and five of them report on various interventions intended to improve the situation. As indicated in Table 4, 12 of the reviewed articles are concerned with employment opportunities while two of them relate to health.

In Nepal, Dhungana (36) found that women with disabilities were discriminated against and excluded from employment. Similarly, Kiani (37) reported that women with disabilities in Cameroon had difficulty in obtaining employment due to discrimination by employers. Also, men with disabilities seemed to experience discrimination. Using survey data from rural India, Mitra and Sambamoorthi (38) found that the employment rates for men without disabilities were higher than for men with disabilities. This difference was not explained by differences in human capital and productivity but may result from discrimination in access to employment and from differential returns to characteristics (38). The situation of men has also been studied in Bangladesh. With an objective to examine the impact of disability on the quality of life, Hosain et al. (39) conducted a survey and identified 162 Bangladeshis with disabilities. Nearly 80% of the men above 12 years of age reported that disability had some negative consequence on their employment, for example, lower salary than non-disabled, forced to change employment and unemployment (39).

These findings are supported by further studies. A comparison of the situations in 15 developing countries by Mitra et al. (40) found that people with disabilities were employed to a statistically significantly less degree than people without disabilities in nine countries. In Afghanistan and Zambia, Trani and Loeb (28) found that people with disabilities had lower access to the labor market. People with cognitive, mental or multiple disabilities were the least likely to work (28). Moreover, from a study in India, Uppal and Sarma (41) reported that disabilities and chronic illnesses have a negative association with the probability of working, the effect being stronger in rural areas.

The status of people with mental and psychosocial disabilities has been studied by Drew et al. (34). They found that denial or restriction of employment rights and opportunities was the second most common rights violation (after ‘exclusion, marginalization, and discrimination in the community’), and the workplace or potential workplace was the third most likely environment in which human rights violations take place (after ‘general community setting in everyday life’ and ‘home and family settings’). More than 60% of the respondents said that their ability to find or maintain a job was adversely affected by their mental or psychosocial disability (34). Similarly, from a comparison of the situations of people without impairments and people with musculoskeletal impairments (MSIs) in Rwanda, Rischewski et al. (42) reported that the latter group was less likely to be employed.

People with disabilities in Afghanistan and Zambia tended to have less access to healthcare services (28).

After these descriptions of the situations of people with disabilities with regard to labor rights, interventions that intend to address the problems in this domain are presented in the following.

Fritz et al. (35) report that as a result of an inclusive poverty-reduction strategy process in Cambodia, contacts were established between community councils and organizations and groups of people with disabilities. These councils then took certain actions, including certification of people with disabilities as poor citizens with free access to health centers, and messages to reduce discrimination against people with disabilities and encourage them to participate in socio-economic activities. Cooperation mechanisms were established between official stakeholders and organizations of people with disabilities. Creating sustainable structures of empowerment was found to be crucial (35).

In Nepal, Dhungana (36) found that women got less access to vocational training programs than men. Men got training in skills that are in high demand and which command good salaries, while women were trained in skills they would not be able to use in their villages, which consequently prevented them from returning home to work (36). This finding is supported by a case study of a community-based vocational rehabilitation (CBVR) program for people with disabilities carried out by Alade (43) in Nigeria. She found that 90% of 155 participants had been gainfully employed. Four features of the program were: an orientation toward skills required locally; a preference for non-formal short-cycle program; a reliance on local markets and resources; and promotion of local participation in planning and implementation (43).

Presenting case studies of corporate social responsibility projects in Bangladesh, Werner (44) notes that one of them targeted women with disabilities and that it increased their employment opportunities by improving their job skills.

In a study of 583 people with hearing or ambulatory impairments in Bangladesh, Borg et al. (45) found that the use of assistive technology was associated with certain labor rights. Compared to people with hearing impairments who did not use hearing aids, users of hearing aids were more likely to report better access to medical care, better physical and mental health, and higher participation in work. However, working status did not differ significantly between users and non-users of hearing aids. In the ambulatory group, people using wheelchairs were less likely to work compared to people not using wheelchairs. After adjusting for the level of physical accessibility to the working place, there were no significant differences in work status between these groups. However, wheelchair users reported a higher level of participation in work. Among wheelchair users alone, it was found that those who had used their wheelchair for a longer period of time were more likely to work (45).

### Business rights

Eleven reviewed articles addressed issues related to business rights. The situation of people with disabilities with regard to these rights is described in eight of them, while measures to overcome related problems are addressed by four articles. Nine articles are concerned with issues of developing one's own talents and four articles address freedom of movement.

Opportunities to develop one's own talents through education are lacking for people with disabilities in many countries. In 14 out of the 15 countries studied by Mitra et al. (40), people with disabilities had completed primary school to a significantly less degree than people without disabilities. From Afghanistan and Zambia, Trani and Loeb (28) reported that people with disabilities have lower access to education, particularly women. Similarly, women with disabilities in Nepal reported little or no education, and, as mentioned above, they also got less from the vocational training that they received (36). In their study in Bangladesh, Hosain et al. (39) found that 41 out of 57 respondents aged 5–30 years had been unable to attend school due to disability or were compelled to leave school (39). Also, Rischewski et al. (42) found that children with MSIs were less likely to attend school. In the North West province of Cameroon, women with disabilities had difficulties in obtaining employment due to lack of education. However, they desired to learn income-generating skills and entrepreneurships and expressed a need for capital to run and maintain businesses (37).

Freedom of movement is limited for people with disabilities. In Nepal, inaccessible transportation services, buildings, and toilets as well as the lack of ramps in public areas were identified as major obstacles to freedom of movement, which in turn prevented them from looking for training and jobs and sometimes keeping a job (36). In Cameroon, women with disabilities found it difficult to move around and reach markets, workplaces, and so on, partly because there was no public or accessible transportation system. In addition, they faced negative attitudes when attempting to use taxis or when using the road (37). Savill et al. (46) identified three types of barriers experienced by people with disabilities when using public transport services in urban areas of four countries, namely structural, social, and psychological barriers.

After these descriptions, measures intended to improve the enjoyment of business rights are reported below.

Following an inclusive poverty-reduction process in Cambodia, commune councils called for people with disabilities to participate in vocational training programs (35). As already mentioned above, one of the corporate social responsibility projects in Bangladesh increased job skills for women with disabilities, which led to better employment opportunities (44). The CBVR program in Nigeria included a revolving loan scheme enabling 15 trainees to purchase equipment and materials for their trades. Due to the general breakdown of the country's economy, most of the beneficiaries experienced difficulties in making regular installments to pay back the loan, which hampered the revolving loan scheme and deprived others from its benefit (43).

The study of Borg et al. (45) in Bangladesh reported that users of hearing aids were more likely to report literacy, higher education and higher participation in school than non-users with hearing impairments. They were also more likely to report higher participation in using public transportation and better ambulatory performance. In the ambulatory group, wheelchair use was associated with better ambulatory performance. There was also a positive relation between physical accessibility and level of participation in work (45).

### Checklist assessment

The extent to which the CRPD addresses the Pillars is summarized in Table 4. The checklist indicates which articles (or parts of articles) of the CRPD address which legal empowerment measure and to what extent. Measures lacking article numbers in their rows were not addressed at all by the CRPD.

Regarding the nine legal empowerment measures for access to justice and the rule of law, the CRPD addresses four measures fully, three partly and two not at all. The CRPD does not address any of the five empowerment measures related to property rights. Four out of seven legal empowerment measures related to labor rights are addressed fully and three partly. Finally, out of nine legal empowerment measures related to business rights, two are addressed fully, four partly and three not at all. In summary, 10 out of 30 empowerment measures recommended by the Report are fully addressed by the CRPD, 10 partly addressed, and 10 not addressed at all.

## Discussion

### Discussion of findings

Legal empowerment is considered to be a process through which poor people become protected and are enabled to use the law to advance their rights and their interests. This study has explored the empirical and normative relevance of the four CLEP Pillars to people with disabilities in low- and lower middle-income countries.

With the exception for property rights, the reviewed articles offer relatively consistent evidence for people with disabilities in low- and lower middle-income countries experiencing problems related to the Pillars, even though not all subdomains were covered. Thus, the empirical findings indicate that several – if not all – problems addressed by the Pillars are relevant to this population. However, it is not possible to determine whether the suggested solutions are relevant, as the reviewed articles provide insufficient evidence for the effects of the recommended legal empowerment measures.

The assessment of the CRPD revealed that two-thirds of the legal empowerment measures put forward in the Report are required by the CRPD to promote, protect, and ensure the enjoyment of all human rights and fundamental freedoms, although only one third of the measures are fully supported by the CRPD. Given the support for a majority of the measures, all of the Pillars except property rights are considered to be normatively relevant.

Suggested legal empowerment measures to address access to justice and the rule of law are relatively well supported by the CRPD. However, the CRPD does not provide explicit support for the use of customary, informal or alternative legal procedures to improve access to justice and the rule of law. Only two empirical studies related to this domain were found, indicating that it is an under-researched area.

Besides lacking normative support from the CRPD, property rights issues for people with disabilities in low- and lower-middle income countries seem to be almost unexplored. Neither explicit descriptions of the situation nor possible intervention measures were found in the reviewed literature. Considering property rights being one of the Pillars, the lack of normative support and empirical evidence was unexpected. However, this may partly be explained by the lack of explicit property-related terms used in the search. Another reason may be that property rights is a complex matter. It is not obvious that private ownership is always better than other alternatives. For example, the benefits of private ownership of land over open-access depend on several factors, such as population density, dangers, and costs for enclosure. Under certain conditions, which take into consideration factors such as production level, returns from investments and economies of scale, a communal property system may be more beneficial than an individual property system. Moreover, recent findings indicate that those who possess at least the rights of proprietorship – if not full ownership – are able to govern and manage their systems more effectively than previously presumed (47).

Another property aspect likely to be of immediate relevance to people with disabilities in low-income countries, as they often belong to the most marginalized group of society, is land grabs of various scales. Although land grabs can have a positive impact on local communities, findings from sub-Saharan Africa indicate that the results are more often negative (48). Considering the links among poverty, health, and disability (2), land grabbing is likely to significantly affect the lives of people with disabilities if they are forced to leave their source of food or income without adequate long-term compensation. No literature addressing this aspect has been found, indicating an immediate need to pay attention to land grab experiences and effects among this vulnerable population.

All suggested legal empowerment measures in the domain of labor rights are addressed by the CRPD. This domain also had the largest body of empirical findings describing the situation of people with disabilities and possible interventions. However, with few exceptions, the identified literature addressed employment and work only. The limited number of articles on health and healthcare may be explained by not including health terms in the search. The reason being that they are probably among the most studied areas with respect to people with disabilities (see e.g. Ref. 3). None of the studies on labor rights were longitudinal.

Most of the legal empowerment measures to support business rights find some normative support in the CRPD, although it is not always as specific. Identified empirical evidence is mainly concerned with education, vocational training, and skills development. Only a few studies attempt to describe the effects of related interventions. There are some studies describing barriers to limitations in enjoying the freedom of movement, while only one study of a possible facilitator was identified. However, it is not a longitudinal study.

Based on this review, certain legal empowerment measures recommended by the Report are supported empirically and normatively. However, the suggested measures go beyond both what the CRPD requires and what is supported by the identified evidence base. The Report has previously been criticized for lack of clarity and unsubstantiated claims (16). Therefore, it remains to be seen if its suggested measures for reducing poverty hold true for people with disabilities in low- and lower middle-income countries, or if other factors will continue to be more important causes of poverty. In addition to general causal factors, the opportunities for poverty reduction may be limited due to more immediate factors, such as stigma, and lack of access to rehabilitation services, assistive technology, and physically and cognitively accessible facilities (3, 49).

Although the CRPD is regarded as having empowered the world's largest minority (23), the recommendations of the Report suggests that implementing only the CRPD would not be sufficient to reduce poverty for this group. In fact, Drew et al. (34) argue that, besides adopting and applying the framework of the CRPD, using a range of evidence-based strategies can help to promote human rights. Although no firm evidence was found through this review, complementing the CRPD measures with legal empowerment measures recommended in the Report may be considered.

Concerning people with disabilities in low- and lower middle-income countries, there appears to be a lack of evidence with respect to their poverty situation and appropriate poverty-reduction strategies. In addition to the findings presented in this review, a recent critical review of the links among disability, poverty, and health concluded that the evidence is strikingly thin (2). Considering the current empirical evidence for the Pillars and recommended empowerment measures found through this literature review, the findings warrant research that clarifies whether all domains are relevant to people with disabilities and explores the effectiveness of suggested and other measures. Recognizing that countries differ geographically, culturally, socially, politically, and so on, studies in various settings are called for. This would be helpful in informing the development and implementation of effective strategies aiming at reducing poverty among people with disabilities in low- and lower middle-income countries, and thereby contribute to sustainable and healthier societies (30).

### Discussion of limitations

The critical review is limited in scope to the databases, search terms and the inclusion and exclusion criteria used. Although the labor and business rights domains of the Pillars include measures related to health conditions and care, social security, and talent development (education), search terms for these measures were not used as they have been studied and reported elsewhere (2, 3, 50–52).

The domains of legal empowerment measures are somewhat overlapping, for example, measures 15 and 27 on freedom of association, which could have caused problems in categorizing articles. But as no reviewed articles addressed freedom of association, such problems did not arise.

It may be argued that certain legal empowerment measures are relevant to domains other than those indicated in Table 4. For example, ensuring freedom of movement is likely to be relevant to labor rights and its measure of access to medical care. However, the operationalization of the Pillars followed the measures devised by the Report.

Due to its inclusion criteria, the review has neither considered publications of related international organizations such as the Global Applied Disability Research and Information Network on Employment and Training (GLADNET), the International Labour Organization and the World Bank, nor has UN country reports been considered.

## Conclusions

The CRPD lends support to a majority of the legal empowerment measures recommended by the Report, which indicates that most of them are relevant from a normative perspective. Considering that the empirical evidence demonstrates that several problems related to the Pillars are experienced by people with disabilities in low- and lower middle-income countries, and that little has been published on solutions to these problems, it may be concluded that the problem areas are relevant while it is uncertain whether the devised measures are of immediate relevance to this population. Further research is required to explore the costs and effects of the proposed measures for reducing poverty and consequently promoting health, particularly in comparison with measures devised by the CRPD. The lack of normative and empirical perspectives on property rights calls for studies of their relevance.
